# Quantitative assessment of a spatial multicriteria model for highly pathogenic avian influenza H5N1 in Thailand, and application in Cambodia

**DOI:** 10.1038/srep31096

**Published:** 2016-08-04

**Authors:** Mathilde C. Paul, Flavie L. Goutard, Floriane Roulleau, Davun Holl, Weerapong Thanapongtharm, François L. Roger, Annelise Tran

**Affiliations:** 1 CIRAD, UPR AGIRs, F-34398, Montpellier, France; 2 IHAP, Université de Toulouse, INRA, ENVT, Toulouse, France; 3 EPIA, INRA, 63122 Saint Genès Champanelle, France; 4 CIRAD, UPR AGIRs, 10900 Bangkok, Thaïland; 5 Kasetsart University, 10900 Bangkok, Thailand; 6 National Veterinary Research Institute, Phnom Penh, Cambodia; 7 Department of Livestock Development, Bangkok, Thailand; 8 CIRAD, UMR TETIS, F-34398, Montpellier, France

## Abstract

The Highly Pathogenic Avian Influenza H5N1 (HPAI) virus is now considered endemic in several Asian countries. In Cambodia, the virus has been circulating in the poultry population since 2004, with a dramatic effect on farmers’ livelihoods and public health. In Thailand, surveillance and control are still important to prevent any new H5N1 incursion. Risk mapping can contribute effectively to disease surveillance and control systems, but is a very challenging task in the absence of reliable disease data. In this work, we used spatial multicriteria decision analysis (MCDA) to produce risk maps for HPAI H5N1 in poultry. We aimed to *i*) evaluate the performance of the MCDA approach to predict areas suitable for H5N1 based on a dataset from Thailand, comparing the predictive capacities of two sources of *a priori* knowledge (literature and experts), and *ii*) apply the best method to produce a risk map for H5N1 in poultry in Cambodia. Our results showed that the expert-based model had a very high predictive capacity in Thailand (AUC = 0.97). Applied in Cambodia, MCDA mapping made it possible to identify hotspots suitable for HPAI H5N1 in the Tonlé Sap watershed, around the cities of Battambang and Kampong Cham, and along the Vietnamese border.

The Highly Pathogenic Avian Influenza (HPAI) H5N1 virus is now considered endemic in several Southeast Asian countries. In Thailand, no outbreaks have been reported since 2008, but surveillance and control are still important to prevent any new HPAI H5N1 incursion. In Cambodia, the virus has been circulating since 2004. So far the country has reported 42 avian outbreaks and 56 human cases (including 39 deaths), with the latest case reported in March 2014^1^. In 2012, the population of Cambodia was 14.86 million people, with 73% of the population depending exclusively on agriculture for their livelihood^2^. The majority of poultry are raised on small backyard farms, often in close contact with other livestock and humans. Biosecurity measures are practically non-existent, exposing the animals to infectious diseases with high levels of mortality^3^. HPAI H5N1 is now considered endemic in the poultry population of Cambodia^4^. With a flock mortality rate above 50%, the disease has had a dramatic effect on the livelihoods of poor farmers^2^. The detection of poultry cases relies almost exclusively on passive reporting by farmers, with occasional active surveillance components funded by external donors^5^,^6^. When HPAI H5N1 outbreaks are confirmed, the current policy in Cambodia is to cull the poultry in the affected village without providing economic compensation. This means that farmers will first try to manage the outbreak locally with the use of disinfectants or by selling their animals to other villages. Reports are often forwarded only when the situation is out of control, delaying the detection of outbreaks and leading to the discovery of HPAI H5N1 in a region with the detection of a human case^7^. In this context, underreporting and thus under-detection of HPAI H5N1 cases in Cambodia is very frequent, making the actual disease distribution in the country poorly known.

Risk maps can effective help highlight areas where surveillance and disease control should be targeted. However, in countries with weak primary health care systems such as Cambodia, their production can be hampered by a lack of reliable disease data. In such situations, knowledge-driven modeling methods, including spatial multicriteria decision analysis (MCDA), have been identified as an alternative to classical statistical approaches^8^. MCDA has been used in studies seeking to assess the suitability of areas for the occurrence of Rift Valley fever^9^,^10^ and African swine fever^9^ in Africa and HPAI H5N1 in Asia^8^. MCDA methods have also been applied in disease-free areas to map the suitability for the emergence of Rift Valley fever in Europe^10^,^11^. Contrary to statistical models which require reliable disease data for the output variable, spatial MCDA approaches are based on decision rules derived from existing knowledge and applied to a set of predictor factors to identify areas potentially suitable for disease^12^. The terms “spatial MCDA”, “spatially explicit MCDA”, and “GIS-based MCDA” have been found interchangeably in the literature^8^,^12^,^13^. Applications of this method to health issues most often assume spatial stationarity of the relationships between attributes and outcome. The methodological steps required in MCDA approaches encompass factor selection, definition of relationships between risk factors and the disease, attribution of weights to factors, combination of risk factors and creation of risk maps, sensitivity analysis, and validation. The three first steps of the analysis can be achieved by extracting information either from a literature review^14^ or by using expert opinion^15^. Whatever the method used, these steps are recognized as potential pitfalls of MCDA approaches^8^,^13^,^16^. Moreover, it has been assumed that knowledge-driven models have inferior predictive capacity than data-driven ones; however, comparative studies of the two approaches are still needed to support this assumption^14^. The validation of maps produced by knowledge-driven models is therefore an essential but challenging task. In epidemiological situations, the validation of MCDA maps is always difficult due to a lack of disease data, and it is frequently limited to a visual comparison with existing data sources^16^. In the field of animal health, to our knowledge only one study has used quantitative methods rather than visual appraisal to assess the correlation between MCDA risk maps and actual disease presence. This study aimed at producing risk maps for HPAI H5N1 in Asia^14^. Although it provided strong evidence of the validity of MCDA approaches to establish risk maps at a continental level, the impreciseness of the source of outbreak data (EMPRES-i) used for validation was an important limitation to this study.

In the present work, we aimed to *i*) evaluate the performance of an MCDA approach to predict suitable areas for HPAI H5N1 based on a dataset from Thailand, comparing the predictive capacities of two sources of *a priori* knowledge (literature and experts), and *ii*) apply the best method to produce a risk map for HPAI H5N1 in poultry in Cambodia.

## Materials and Methods

ArcGIS (version 10.0; ESRI, Redlands, CA) and IDRISI (Clark Labs, Worcester, USA) software were used to manage geoprocessing and implement the model. R software version 3.2.4 (R Foundation for Statistical Computing, Vienna, Austria) was used for sensitivity analysis and map validation.

A common set of 10 risk factors was selected for Thailand and Cambodia. Of the 10 factors, 3 correspond to poultry densities. Two types of duck farming systems were considered as they are known to increase HPAI risk: (i) free-grazing ducks, which roam freely in rice fields and can be moved over large distances^17^, and (ii) farm ducks, raised under low biosecurity systems with frequent movements of farmers and workers^18^. Although only a weak association had been previously observed^14^,^19^, backyard chicken density was introduced in the model as low biosecurity levels may contribute to HPAI H5N1 spread^20^,^21^. We also considered the proportion of surface covered with open water^22^, as well as two factors describing rice cultivation and subsequent flooding (proportion of surface covered with rice fields and mean number of rice crops)^22^,^23^. Water may facilitate oro-faecal transmission between hosts as the HPAI H5N1 virus can remain infective several days in water even at the temperatures encountered in Asia^24^. Three anthropogenic risk factors (density of human population, road density, proximity to main cities) which have been previously identified with greater HPAI H5N1 risk^17^,^18^,^22^,^25^,^26^ also were included. They are surrogates for poultry markets and areas of intensive poultry trading activities, which may increase HPAI transmission through flows of contaminated poultry or fomites. Although it cannot be considered as a causal factor, altitude was introduced in the model as it has been repeatedly found associated with HPAI in previous research^19^, and it is a correlated with several variables (such as irrigation networks, or contact between farms) involved in the transmission of HPAI.

### Experts survey

Experts were selected among researchers, academics, and staff at non-governmental, governmental and international organizations with recognized experience on the risk factors associated with HPAI H5N1. Fourteen experts were contacted in Thailand and eleven in Cambodia. A questionnaire was developed using SurveyMonkey™ and submitted online. The questionnaire consisted of three steps. First, experts were asked to select a set of relevant risk factors from a list of 10 putative factors. Second, they had to choose a relationship between each selected factor and the risk of HPAI H5N1. Four types of relationships were proposed: linear, sigmoidal, quadratic (with highest risk associated with a specific threshold), and linear bi-directional, with a linear increase of the risk increasing until a first threshold, then constant and decreasing after a second threshold. For non-linear relationships, experts could choose thresholds corresponding to “very low”, “low”, “intermediate”, “high”, “very high” values of the explanatory factors as derived from quantiles. To assist in that process, we used influence diagrams as graphical aids^27^ which illustrated the relationship between factors and risk of HPAI H5N1. Third, experts had to fill in a pair-wise comparison matrix used in the analytical hierarchy process (AHP), where each factor was compared with the others, relative to its importance, on a nine-point scale ranging from 1/9 (“extremely less important”), through 1 (“equal importance”), to 9 (“extremely more important”)^28^.

### Data collection and geoprocessing of HPAI H5N1 risk factor layers

The density of free-grazing ducks, farm ducks, and backyard chickens was calculated at a subdistrict level (third-level administrative division, mean area 70 km^2^) in Thailand, using a dataset obtained from the 2005 Census made by the Department of Livestock Development (DLD). In Cambodia, similar factors were computed at a commune level (also a third-level administrative division, mean area 112 km^2^) using a census made by the National Veterinary Research Institute in 2010. The proportion of surface covered with rice fields and the mean number of rice crops were calculated in a 2-km radius around each pixel for the two countries from a time series of MODIS images collected in 2005 using methods described elsewhere^29^,^30^. The proportion of surface covered with open water for the same radius was calculated from a vector map obtained on DIVA-GIS (http://www.diva-gis.org/), as well as the density of human population (from the LandScan™ High Resolution Global Population raster dataset, http://web.ornl.gov/sci/landscan/, 1-km resolution) and the mean altitude (SRTM, Shuttle Radar Topography Mission, http://srtm.csi.cgiar.org/SELECTION/inputCoord.asp, 90-m resolution). A ‘proximity to main cities’ layer was obtained from a raster map generated from locational point data corresponding to cities of >100,000 inhabitants in Thailand^18^ and provincial capitals in Cambodia. All predictors were transformed into raster layers with a pixel size of 90 m × 90 m.

### Standardization of geographical layers and weights attribution for experts maps

To make comparisons possible, layer values were standardized to a common scale (ranging from 0, unsuitable, to 1, totally suitable) by using fuzzy membership functions corresponding to the relationships selected by experts, resulting in one standardized factor layer per expert. For each risk factor, the average of these layers was then calculated to obtain a single standardized layer.

Using the principal eigenvector of the pair-wise comparison matrix filled in by each expert, a best-fit of weights was calculated for each expert and factor^13^. The average of individual weights attributed by experts was then calculated to obtain a single weight for each factor. Finally, to take into account possible collinearity between the risk factors, the Pearson correlation coefficient was determined for each pair of factors. If it was greater than 0.4, the weights of both factors were decreased by 10%, and the weights of all other risk factors were increased proportionally with their original ratio^14^.

### Standardization of geographical layers and weights attribution for literature-based maps

In order to explore the performance of two possible sources of *a priori* knowledge (literature vs experts), a literature-based map was generated in Thailand. The same steps used to construct the expert-based maps were followed. We relied on a previous study which had extracted from the literature weights and membership functions for six risk factors (waterfowl density, chicken density, human population density, proximity to roads, proximity to water, and proximity to rice) to produce H5N1 suitability maps in Southeast Asia^14^. We then applied the weights and relationships presented in Table 1 to the geographical layers we gathered in Thailand.

### Generation of final suitability maps

A weighted linear sum was then applied to risk factor data layers using the final weights previously calculated (Equation 1).
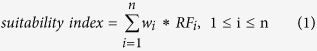
where *n* is the number of risk factors, *w*_*i*_ the weight and *RF*_*i*_ the value of risk factor *i*.

This made it possible to compute a suitability index for HPAI H5N1 in poultry at a 90-m resolution. Three maps were produced: 2 expert-based maps (in Thailand and Cambodia) and 1 literature-based map (in Thailand only).

### Uncertainty and sensitivity analysis

A sensitivity analysis was conducted to assess the sensitivity of the method to the weights assigned by experts to the different factors.

For each risk factor, the range of weight deviations was defined by the minimum and the maximum values given by the different experts. Ten weight values (*w*_*m*_), uniformly distributed throughout this range, were tested, adjusting the weights of the other risk factors (*w*_*i*_) so that the sum of all of the weights was equal to one (Equation 2).
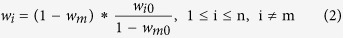
where *w*_*m0*_ and *w*_*i0*_ are the weights in the base model of the main changing risk factor and of the *i*-th risk factor, respectively.

For each combination of weights obtained, a map of suitability index for HPAI H5N1 in poultry was computed according to Eq. 1; a total of 100 and 90 maps were thus generated for Thailand and Cambodia, respectively.

Based on these different outputs, the contributions of the variation weights to the suitability index variability were evaluated using a linear regression^31^. We considered outputs at different scales: the change in the suitability index *i*) averaged at the country-level in order to identify the most sensitive weights and *ii*) averaged at the local level (sub-district in Thailand, commune in Cambodia), to visualize the spatial pattern of weight sensitivities, which may be spatially heterogeneous^32^,^33^. For each aggregated output, a linear regression model was fitted with all of the principal effects of the risk factors. The contribution of factor *i* to the variation in output was the ratio of the sum of squares related to *i* on the total sum of squares of the model. At the local level, the dominating weight was identified for each geographical unit.

Moreover, an uncertainty surface was produced for the two countries, representing the standard deviation of the different suitability maps resulting from the changes in weights^33^.

### Map validation

The predictive ability of the MCDA models was quantitatively evaluated by calculating the Area Under the Curve (AUC) from ROC analysis (Supplementary Information, section S1). Predicted values were obtained from the suitability maps by calculating the average risk in each subdistrict (Thailand) and in each district and commune (Cambodia). Observed values corresponded to presence/absence data for HPAI H5N1 at the same aggregation level. In Thailand, outbreak data were obtained from the DLD. Out of 7366 subdistricts, 767 had been infected by HPAI H5N1 from July 2004 to May 2005^18^. It has been shown that the surveillance system had a good sensitivity at that time^34^. In Cambodia, poultry outbreaks of H5N1 are under-detected by the national surveillance system^2^. Validation of MCDA maps was thus carried out instead using fine-scale data obtained from an outbreak investigation study^35^,^36^ described in Supplementary Information, section S2.

## Results

### Weights attributed by experts (Thailand and Cambodia)

After the elimination of missing or incomplete responses, data from 7 and 5 experts were retained in Thailand and Cambodia, respectively. In both countries, the experts attributed the greatest weight to the density of free-grazing ducks factor (Table 2). Next in terms of influence, the experts ranked density of farm ducks, proportion of rice fields and number of rice crops in a 2-km radius.

### Suitability maps

**Figure 1:**
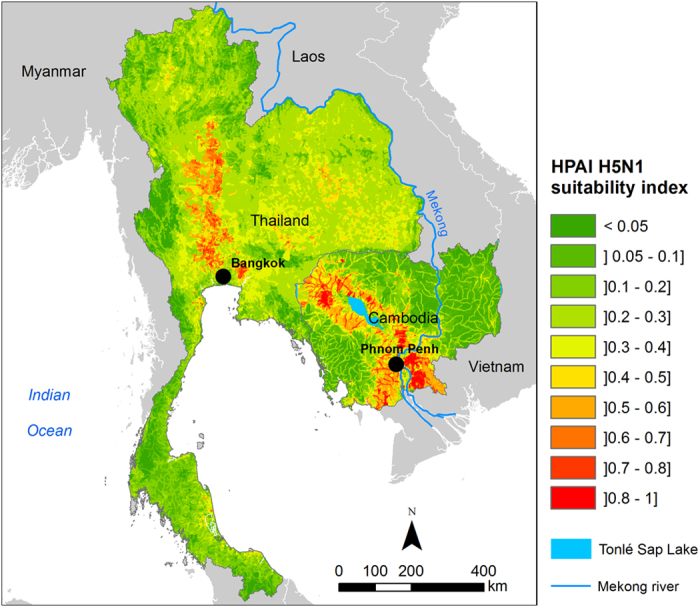
Suitability map for occurrence of Highly Pathogenic Avian Influenza (HPAI) H5N1 in domestic poultry in Thailand and Cambodia. Maps were generated using ArcGIS (version 10.0; https://www.arcgis.com/features/).

The suitability for occurrence of HPAI H5N1 in domestic poultry was displayed on a continuous scale ranging from 0 (least suitable) to 1 (highly suitable) (Fig. 1). In Thailand, the most suitable areas were located in a corridor extending along the Central Plain. Risk hotspots of limited extent were also identified west of Bangkok and in the North East region. In Cambodia, the MCDA model pointed out a higher risk of HPAI H5N1 in the surroundings of Tonlé Sap Lake, around Phnom Penh–the capital city, as well as in areas of the lower Mekong basin bordering Vietnam.

### Uncertainty and sensitivity analysis

**Figure 2:**
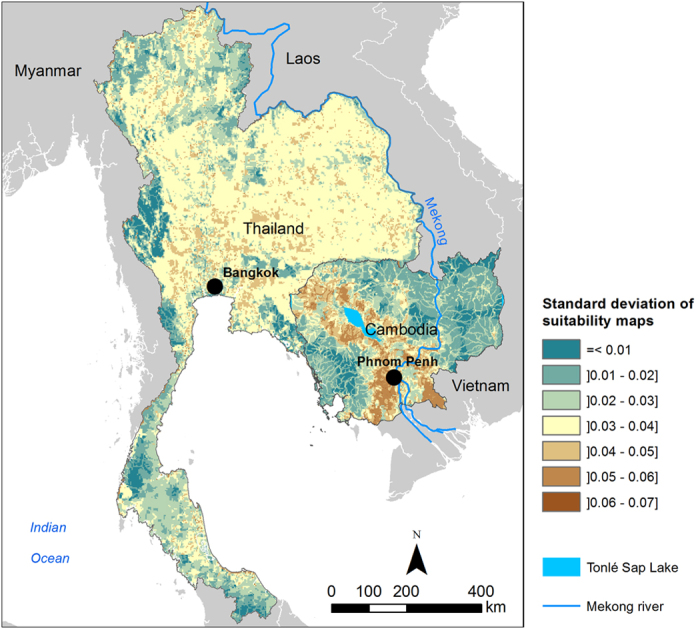
Uncertainty map (standard deviation of the suitability maps for HPAI H5N1 in domestic poultry in Thailand and Cambodia). Maps were generated using ArcGIS (version 10.0; https://www.arcgis.com/features/).

The uncertainty surface produced for Thailand and Cambodia (Fig. 2) showed that the predicted risk areas for H5N1 occurrence according to the suitability index are robust, meaning that they remain stable when risk factors weights are varied. The maximum standard-deviation value (STD) is less than 0.1. Results highlighted a spatial heterogeneity in uncertainty, with higher uncertainty in high H5N1 suitability areas.

**Figure 3:**
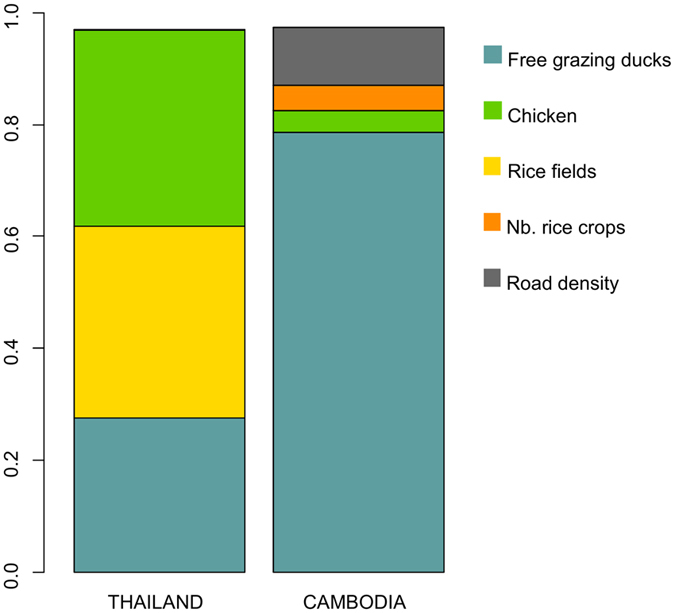
Contribution of risk factor weights to model output variance at the country level, in Thailand and Cambodia.

At the country level, the variation in suitability index was mainly explained by three of the ten risk factor weights for Thailand and four of the nine risk factor weights for Cambodia (Fig. 3). In Thailand, the weight of free-grazing ducks density, proportion of rice fields, and chicken density were the most sensitive parameters. In Cambodia, the weight of free-grazing ducks density was the most sensitive parameter (contributing to 78% of output variance), and, to a lesser extent, the weight of road density, number of rice crops, and chicken density.

**Figure 4:**
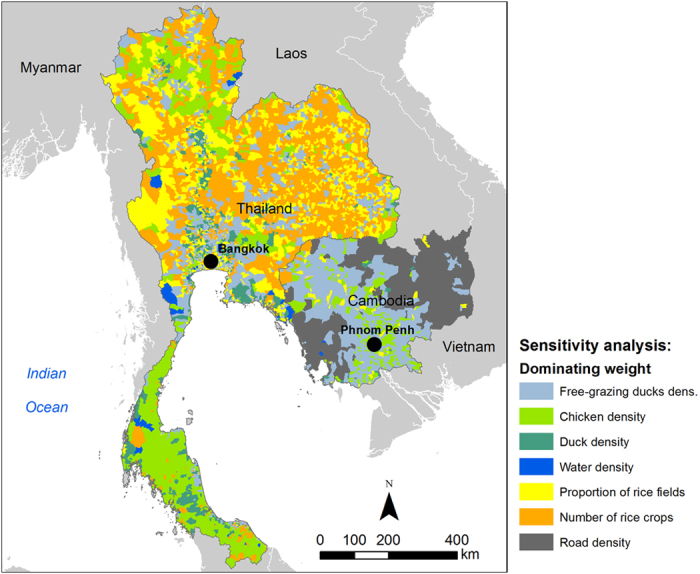
Impact of risk factor weight on suitability index variability for HPAI H5N1 at a local level, in Thailand and Cambodia. Maps were generated using ArcGIS (version 10.0; https://www.arcgis.com/features/).

The sensitivity analysis performed at the local level (sub-district and commune level for Thailand and Cambodia, respectively) showed that the “dominating weight”, that is, the risk factor weight whose variation has the higher impact on the suitability index variability, varied over space in Thailand and Cambodia (Fig. 4). In Cambodia, the weight attributed to free-grazing ducks density dominated around Tonlé Sap Lake and Mekong River, whereas in the rest of the country the weight attributed to road density was the most sensitive one. In Thailand, the weight of free-grazing ducks density was predominant around Bangkok, but results showed that rice-related risk factors (proportion of rice fields, number of rice crops) had the most sensitive weights in most of the northern districts. In the south, the weight of the “chicken density” factor was predominant. Full details on the variations of the suitability index with weights of the different risk factors are given in Supplementary Information, section S3.

### Map validation

Results showed that the expert-based model had an excellent prediction capacity in Thailand (AUC = 0.97, CI 95: 0.96–0.97), which was significantly higher than the literature-based model (AUC = 0.74, CI 95: 0.72–0.76). The predictive capacity of the expert-based model in Cambodia was higher at the district (AUC = 0.72, CI95: 0.59 – 0.85) than the commune level (AUC = 0.65, CI95: 0.61 – 0.70).

## Discussion

The limitations of MCDA for mapping have been presented and discussed extensively elsewhere^8^,^13^. They include double counting issues associated with correlated factors, which were tackled in the present study by following a method presented in previous work^14^. The subjectivity associated with experts’ choices in the selection of risk factors, membership functions and weights also has been identified as an important limitation of MCDA. Experts’ opinion has been widely used in analytical frameworks which require the incorporation of *a priori* knowledge, such as probabilistic risk assessment^37^ and Bayesian modeling^38^. Although there is no absolute guideline on which to base the number of experts to be invited for eliciting health issues^39^, the number of experts we consulted is in line with sample sizes previously reported^40^. Furthermore, the opinion of experts on risk factors for HPAI H5N1 may vary depending on their experience and the type of organization they belong to (research institute, governmental health services …). The influence of experts’ background on individual opinions would worth being investigated in further works, provided the number of experts is large enough to support meaningful statistical analysis. The aggregation of opinions is also an important step in the elicitation process^40^. In this study, experts’ opinion was collected in an individual manner using an electronic questionnaire and aggregated using a mathematical approach based on weighted linear combination. Other options, including behavioral approaches, are also available for aggregating experts’ knowledge and it would be worthwhile to explore these in further works. These approaches aim at producing some type of group consensus among experts, who are typically encouraged to share their assessments in an anonymous environment for Delphi techniques^41^ or in direct interaction with other experts with Nominal Group methods^42^. Behavioral approaches have been previously applied to spatial MCDA^15^,^43^, but they remain rare. The integration of behavioral experts’ elicitation methods in MCDA risk mapping could make the process of prioritizing disease surveillance and control more inclusive and participatory.

Despite all of the drawbacks associated with spatial MCDA, the quantitative validation of the risk maps produced for HPAI H5N1 in Thailand showed that the predictive performances of these models were reasonable to very good. When comparing the two possible sources of *a priori* knowledge, we found that maps produced by local experts’ opinion resulted in significantly higher predictive power (AUC = 0.97, [0.96–0.97]) than those produced from a literature review (AUC = 0.74 [0.72–0.76]). This may partly be explained by discrepancies in factors ranking between experts and literature. Both knowledge sources identified waterfowl as the most influential factor. However, when it came to secondary factors, the literature identified human population density while experts’ opinion identified farm ducks and rice-related factors. Furthermore, and in contrast with literature data, the weight attributed by experts to human population density was considerably lower than the weight attributed to poultry density, rice cultivation, and water coverage. Another possible explanation for the very good performance of the expert-based model in Thailand is that some of the experts had previous experience in statistical modeling of HPAI H5N1 risk factors. This may have influenced the results. While spatial MCDA models are believed to have poorer predictive power than statistical models, it is noteworthy that the expert-based model developed in the present study had greater predictive capacity than previously published statistical models^23^. The spatial pattern of discrepancies and concordances between maps produced from these two approaches would worth being explored in further works, as suggested by a previous study comparing statistical and expert-based modeling^44^.

In Cambodia, the predictive capacity of the expert-based model can be considered as reasonable (AUC = 0.72, [0.59–0.85]), although it is lower than that of the Thailand model. There may be three explanations for this observation. First, it could be due to an insufficient resolution of poultry data in Cambodia, in particular regarding free-grazing ducks density which was the most sensitive parameter in the country. Although poultry data was collected at a similar (3^rd^) administrative level, the resolution was coarser in Cambodia than in Thailand (the average area was 112 km^2^ for communes in Cambodia vs 70 km^2^ for Thailand subdistricts). Second, it is also possible that experts’ weighting in Cambodia was finally less relevant regarding HPAI outbreak location than in Thailand. This could be explained by the fact that experts in Cambodia had to attribute weights without being able to rely on previous statistical models (as no spatial statistical models for HPAI H5N1 have yet to be developed in Cambodia), and in the absence of precise knowledge of the spatial location of previous HPAI outbreaks (as outbreaks reported in OIE/WHO databases are too sparse to have a clear picture of the actual spatial pattern of H5N1). Third, it is possible that the epidemiological mechanisms for HPAI H5N1 persistence in Cambodia, where the disease is endemic, differ slightly from those observed in Thailand, which have been extensively studied in an epidemic situation. Moreover, current outbreaks in Cambodia are only caused by the clade 1 virus, found solely in the southern Mekong basin. A new endemic virus clade (1.1A) has even been identified in Cambodia^4^. This may have rendered the experts’ task of attributing weights and relationships especially challenging for Cambodia.

While HPAI H5N1 has been circulating widely in Cambodia since 2004, the origin and dynamics of these epizootics in the country remain unclear^4^. Contrary to other Southeast Asian countries such as Thailand^17^,^18^,^26^, Vietnam^23^,^45^,^46^, and Indonesia^25^, the spatial pattern of HPAI H5N1 has been poorly explored in Cambodia to date. Risk maps which were produced at a regional level in Southeast Asia^14^,^23^ provide interesting insights for Cambodia, but their spatial resolution remain relatively coarse (500-m to 1-km). In the present work, a spatial MCDA approach applied to an original dataset of explanatory factors made it possible to produce a risk map for HPAI H5N1 in poultry in Cambodia at the very fine resolution of 90-m.

In Cambodia, a comprehensive and quantitative map validation was not possible due to the incompleteness of HPAI H5N1 data in this country. However, validation of the map with outbreak data collected in the two provinces gave fair results at the district level (AUC = 0.72, CI95: 0.59–0.85). Moreover, the SA demonstrated that that the predicted risk areas for HPAI H5N1 occurrence according to the suitability index are robust. This suggests that the MCDA map we produced provides valuable insight into the epidemiological situation of HPAI H5N1 in poultry in Cambodia. The expert-based MCDA model pointed out a higher risk of HPAI H5N1 in the Tonlé Sap watershed, with a HPAI H5N1 suitability index of more than 0.5. The Tonlé Sap Great Lake and floodplains constitute the largest continuous areas of natural wetland habitats remaining in the Mekong system, while being the largest permanent freshwater body in Southeast Asia^47^. The extensive wetlands born from hydrological cycles are home to a high level of biodiversity and the site is considered to be of great ecological significance^48^. The landscapes of the Tonlé Sap watershed are characterized by a predominance of marshes and rice fields. This type of agro-ecosystem is known to be favorable to H5N1 virus persistence in water and mud. Wetland-rice agro-ecosystems have repeatedly been found associated with HPAI outbreaks in various Southeast Asian countries^25^,^49^, as well as with low pathogenic avian influenza in Madagascar^50^. In addition to this favorable agro-ecosystem, additional risk factors may contribute to increasing the risk for HPAI H5N1 in localized hotspots in Cambodia. In the north-western part of the floodplain, the high population density found in Battambang province is associated with intense poultry trading and marketing activities^51^. This may support an increased risk of HPAI H5N1 transmission in this area, either through the marketing of infected and apparently healthy poultry, or through contaminated fomites^19^. The high suitability index for HPAI that we identified in Kampong Cham, located at north of Phnom Penh and on the Mekong River, also may be due to the intensity of duck production and trading networks which are recognized as being of national importance^51^. Indeed, it is known that a large proportion of domestic ducks can become infected with HPAIV H5N1 without showing apparent clinical signs^52^ and can spread the virus without being noticed. In addition to factors contributing to HPAI maintenance, the lower part of the watershed (including Prey Veng, Takeo and Kampot provinces) is also exposed to increased risk for HPAI introduction given its proximity to the Vietnamese border. In these provinces, cross-border movements of free-grazing ducks are common, with flocks often coming together. Epidemiological links between these two countries have already been established regarding HPAI H5N1^53^. Despite the vaccination programs implemented in Vietnam against HPAI H5N1, there is still some silent circulation of the virus in Vietnamese duck flocks due to inefficient vaccine coverage^54^. Close contact with the immunologically naïve population in Cambodia may be revealing the disease. Previous virological studies suggest that two distinct epidemiological mechanisms may co-exist in Cambodia^55^. The presence of lineage 6, endemic to Cambodia only, indicates the existence of a transmission specific to this country whereas the presence of lineage 5 in both Cambodia and Vietnam indicate a repeated source of reintroduction of the virus^4^, possibly associated with cross-border poultry movements. Moreover, these three provinces have large urban markets where a large number of ducks introduced illegally from Vietnam are sold. These markets are almost totally composed of sellers with high surpluses, known to be at higher risk of maintaining the H5N1 virus^51^,^56^. The incorporation of detailed information on the geographical location of poultry H5N1 isolates into future analyses would certainly contribute to a better understanding of the epidemiological mechanisms which underlie the spatial pattern of HPAI H5N1 circulation in Cambodia.

In settings with very limited financial and human resources, risk-based surveillance approaches have been identified as a way to set surveillance priorities, select appropriate surveillance activities and enable an optimal allocation of resources^2^ with higher benefit-cost ratios^57^. In this perspective, spatial information can be incorporated in the decision-making process to define risk strata or to select areas where the consequences of diseases will be most critical^57^. In Thailand, the active component of the HPAI H5N1 surveillance system has been targeted on “high risk areas”, defined by the DLD according to the location of free-grazing ducks and previous HPAI outbreaks. It has been proved that this spatial risk-based strategy resulted in a considerable increase in the sensitivity of the whole surveillance system^34^. In Cambodia, results from the present study could thus contribute, in a similar way, to identifying high risk areas in order to strengthen surveillance and control in poultry and, in turn, prevent the occurrence of human H5N1 cases. Specific surveillance activities in restricted areas, such as sentinel duck flocks or participatory surveillance in sentinel villages^6^, should enable a more cost-effective design. These high risk areas also could be selected for the implementation of One Health surveillance where joint decisions about planning, execution and budget could be taken. It has been shown that One Health surveillance could increase human case detection from 57% to 93%^58^. Risk-based surveillance also could be modulated according to the month, targeting seasonal holidays (e.g., Khmer New Year) during which markets experience great increases in their poultry population and human contacts^59^.

## Conclusion

The results of this study, which used a Thailand dataset to compare the performance of literature and experts as sources of *a priori* knowledge for spatial MCDA, showed that the expert-based model had a very high predictive capacity. This suggests that expert opinion can be used to produce risk maps in situations where disease data are scarce, i.e. under-reporting of outbreaks for epidemic/endemic diseases or quasi-absence of outbreaks in the case of emerging infectious diseases. Applied in Cambodia, expert-based MCDA risk mapping made it possible to identify hotspots suitable for HPAI H5N1 in poultry. The Tonlé Sap watershed was identified as an area exposed to high HPAI H5N1 risk, with localized hotspots around the cities of Battambang and Kampong Cham, as well as in provinces located in the lower part of the basin bordering Vietnam.

Despite several drawbacks, MCDA has the advantage of being simpler to implement than statistical models, and thus can be transferred to field stakeholders engaged in disease management. MCDA could thus be seen as a discussion and guidance tool for policy-makers, and could serve as a starting point to define risk-based surveillance and control programs.

## Additional Information

**How to cite this article**: Paul, M. C. *et al*. Quantitative assessment of a spatial multicriteria model for highly pathogenic avian influenza H5N1 in Thailand, and application in Cambodia. *Sci. Rep.* **6**, 31096; doi: 10.1038/srep31096 (2016).

## Supplementary Material

Supplementary Information

## Figures and Tables

**Table 1:** Weights and relationships extracted from the literature-based MCDA by Stevens *et al*. 14 and applied to the Thailand dataset.

	Weight	Relationship between the factor and HPAI H5N1 suitability
Density of waterfowls	0.3768	Sigmoidal, monotonically increasing relationship between 0 and 1000 heads/km^2^ with constant risk thereafter.
Density of chickens	0.0385	Quadratic relationship with highest risk associated with medium density of chickens (500–5000 heads/km^2^), and lowest risk associated with both low (0–500 heads/km^2^) and high (>5000 heads/km^2^) chicken densities.
Human population density	0.2472	Positive linear relationship
Proximity to roads	0.1574	Sigmoidal, monotonically decreasing relationship with greatest risk within 0–5 km of a road, decreasing risk thereafter and negligible risk after 60 km.
Proximity to water	0.1149	Highest risk close (0–5 km) to open water and thereafter decreased in a sigmoidal, monotonic fashion with negligible risk after 10 km
Proximity to rice	0.0652	Highest risk close (0–5 km) to areas suitable for rice growing and thereafter decreased in a sigmoidal, monotonic fashion with negligible risk after 10 km

**Table 2:** Weights attributed by the experts.

	Thailand	Cambodia
Density of free-grazing ducks	0.389 [0.19–0.5]	0.394 [0.06–0.79]
Density of farm ducks	0.090 [0–0.28]	0.080 [0–0.20]
Density of backyard chickens	0.060 [0–0.21]	0.085 [0–0.24]
Proportion of rice fields in a 2-km radius	0.076 [0–0.30]	0.102 [0–0.37]
Number of rice crops in a 2-km radius	0.261 [0–0.5]	0.107 [0–0.17]
Density of free water in a 2-km radius	0.079 [0–0.16]	0.124 [0–0.17]
Road density in a 2-km radius	0.013 [0–0.05]	0.030 [0–0.15]
Density of human population	0.013 [0–0.05]	0.040 [0–0.12]
Proximity to main cities	0.013 [0–0.05]	0.030 [0–0.15]
Altitude	0.003 [0–0.03]	0

Minimum and maximum values are indicated in brackets. A zero value means that the variable was not selected as relevant risk factor by some experts.
