# Supplementary material for: Quantitative assessment of a spatial multicriteria model for highly pathogenic avian influenza H5N1 in Thailand, and application in Cambodia
Source: Sci Rep. 2016 Aug 4;6:31096. doi: 10.1038/srep31096 (PMC4977984; doi:10.1038/srep31096)
Supplement: Supplementary Information [file srep31096-s1.pdf]

Quantitative assessment of a spatial multicriteria model for highly pathogenic avian influenza H5N1 in Thailand, and application in Cambodia

Mathilde C. Paul[1,2,3*], Flavie L. Goutard[1], Floriane Roulleau[1,2], Davun Holl[4], Weerapong Thanapongtharm[5], François L. Roger[1], Annelise Tran[1,6]

[1] CIRAD, UPR AGIRs, F-34398, Montpellier, France

[2] IHAP, Université de Toulouse, INRA, ENVT, Toulouse, France

[3] EPIA, INRA, F-63122 Saint Genès Champanelle, France

[4] National Veterinary Research Institute, Phnom Penh, Cambodia

[5] Department of Livestock Development, Bangkok, Thailand

[6] CIRAD, UMR TETIS, F-34398, Montpellier, France

* Corresponding author:

E-mail : m.paul@envt.fr

Supplementary information, section S1

The predictive ability of the MCDA models was quantitatively assessed by calculating the Area Under the Curve (AUC) from ROC analysis. This method has been widely used for assessing performance of modelling techniques that use binary data as dependent variables[1]. Comparing the binary transformed predicted probabilities obtained from the model (i.e. the suitability index in this work) with the validation presence–absence (i.e. outbreak) dataset makes it possible to estimate the sensitivity (correctly predicted positive fraction, i.e. the percentage of pixels considered infected according to outbreak data and predicted as such by the MCDA model) and specificity (correctly predicted negative fraction, i.e. the percentage of disease-free pixels predicted as such by the MCDA model) [2].

The ROC curve plots the sensitivity against 1-specificity for an entire range of probability thresholds. The calculation of the area under this curve (AUC, which ranges from 0 to 1) provides a single-number discrimination measure across all possible ranges of thresholds. A random probability map produces a diagonal ROC curve, and an AUC value of 0.5. An AUC between 0.5 and 0.7 indicates a poor discriminative capacity of the model, whereas AUC values from 0.7-0.8, 0.8-0.9 and >0.9 correspond to acceptable, excellent and outstanding discrimination, respectively, by the model [3].

Supplementary information, section S2

Between February and April 2010, two HPAI H5N1 outbreaks (in Prolay Meas village, Takeo province, and in Peam Sdey village, Prey Veng province) were reported to the Department of Animal Health and Production of Cambodia. Following the first report, an investigation team was sent to every village within a 20 km radius of the village where the first case was reported and laboratory confirmed to look actively for HPAI H5N1 clinical signs of disease in poultry. HPAI H5N1 cases were detected using inclusion criteria based on mortality level and clinical signs [4,5]. HPAI H5N1 was detected on poultry in 115 out of 209 villages in Takeo province, and in 39 out of 229 villages in Prey Veng province.

Supplementary information, section S3

Variations of HPAI H5N1 suitability index with risk factor weights in Thailand (a) and Cambodia (b). For each considered weight, a box-and-whisker diagram graphically depicts the maximum, minimum, median, lower and upper quartiles values of the model output obtained from the simulations with ten different levels of the risk factor weight tested (expressed as the difference to the weight value used in the final model).

a

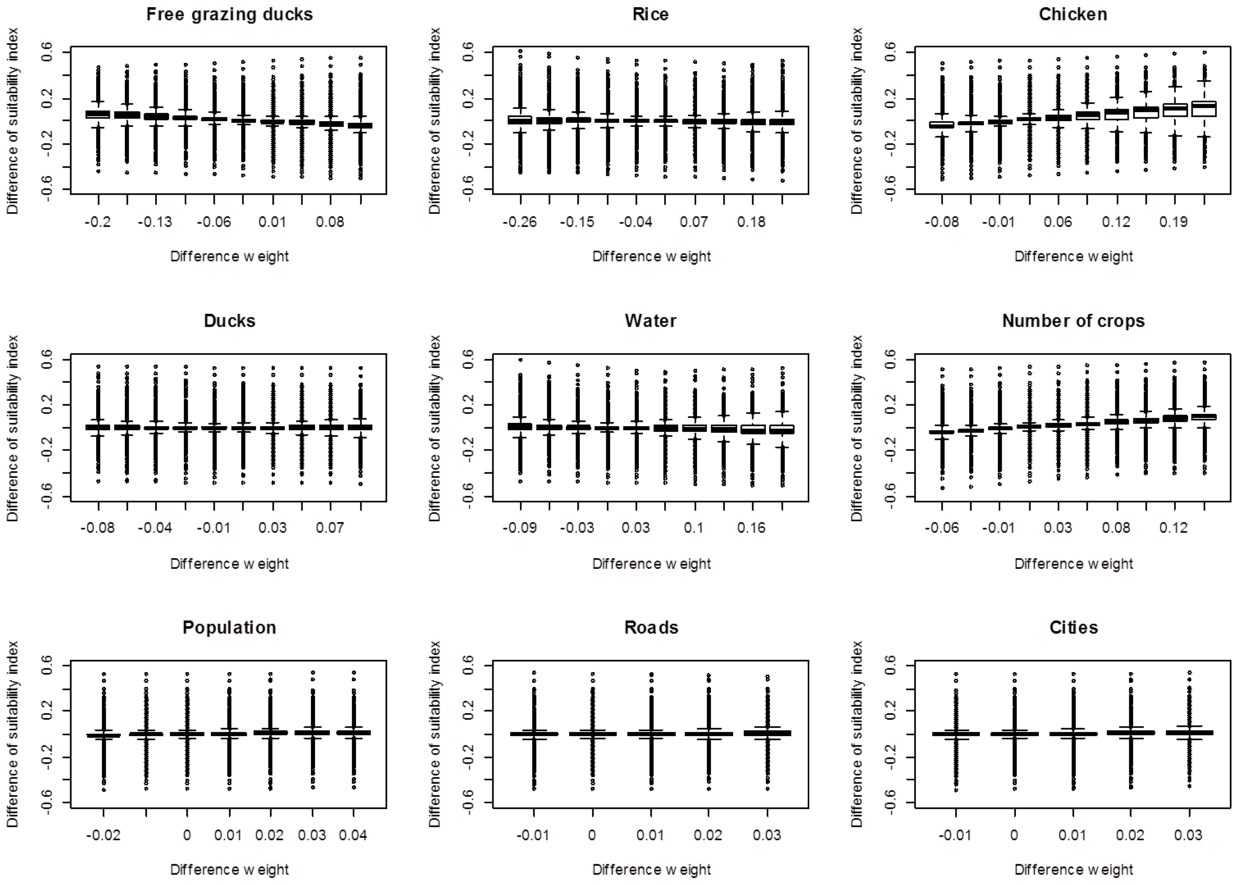

b

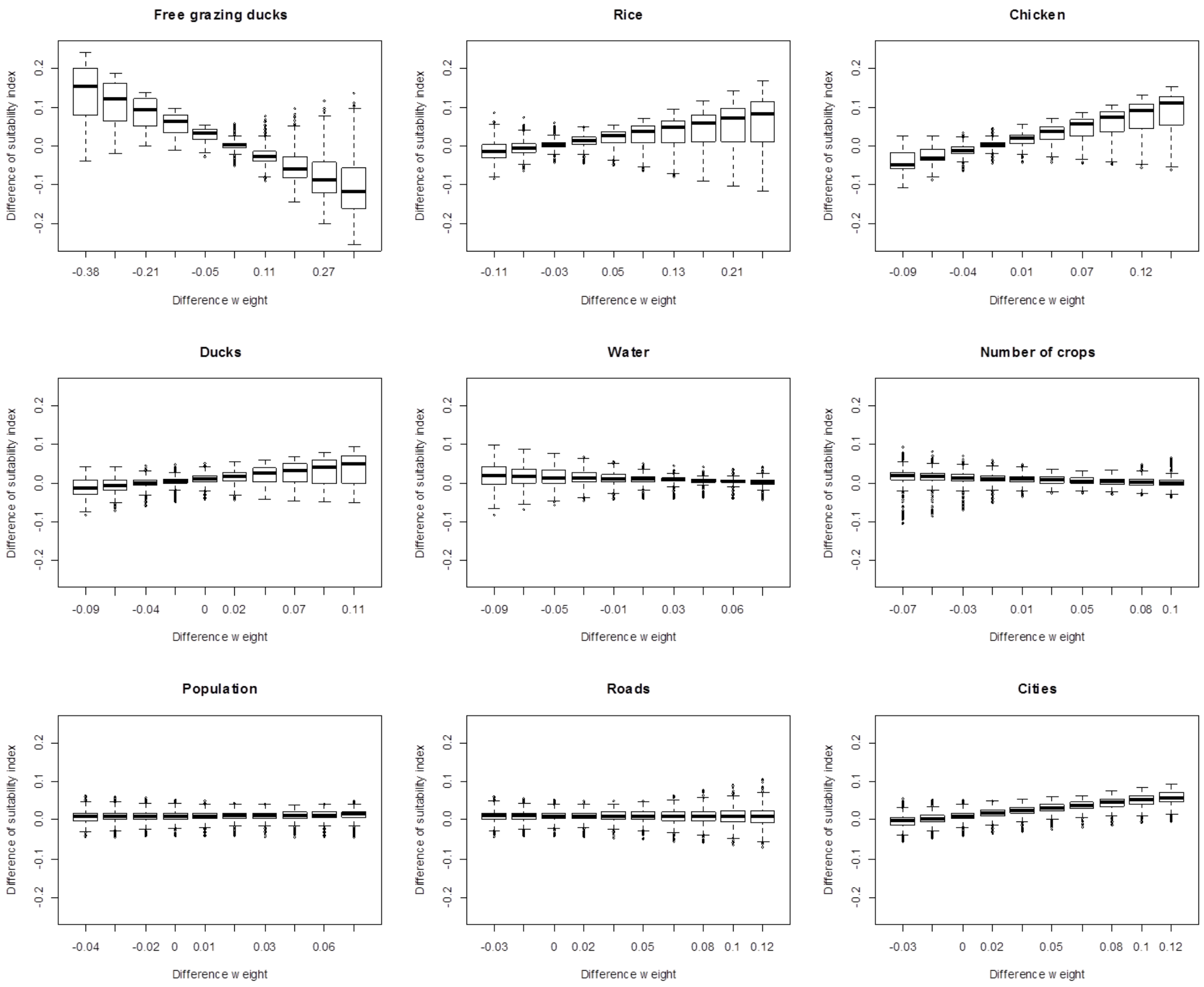
Free grazing ducks
Rice
Chicken
Ducks
Water
Number of crops
Population
Roads
Cities
Difference of suitability index
Difference w eight
0.2
0.1
0.0
-0.1
-0.2
-0.38 -0.21 -0.05 0.11 0.27
-0.11 -0.03 0.05 0.13 0.21
-0.09 -0.04 0.01 0.07 0.12
-0.09 -0.04 0 0.02 0.07 0.11
-0.09 -0.05 -0.01 0.03 0.06
-0.07 -0.03 0.01 0.05 0.08 0.1
-0.04 -0.02 0 0.01 0.03 0.06
-0.03 0 0.02 0.05 0.08 0.1 0.12
-0.03 0 0.02 0.05 0.08 0.1 0.12

Supplementary information, references

1. Brooker, S., Hay, S.I., Bundy, D.A. Tools from ecology: useful for evaluating infection risk models? *Trends. Parasitol.* **18**, 70-74 (2002).
2. Lobo, J. M., Jiménez-Valverde, A., Real, R. AUC: a misleading measure of the performance of predictive distribution models. *Global Ecol. Biogeogr.* **17**, 145-151 (2008).
3. Hosmer, D. W., Lemeshow, S. *Applied logistic regression, Second edition.* Wiley (2000).
4. Conan, A., Holl, D., Goutard, F., Buchy P., San S., Vong S. Clinical definition of highly pathogenic avian influenza (H5N1) outbreaks in Cambodian backyard flocks. Presented at the International Conference on Options for the Control of Influenza VII, Hong Kong SAR, China. (2010, September 3-7).
5. Goutard, F.L. *et al*., Spatio-temporal analysis of avian influenza H5N1 outbreaks in human and poultry population in Cambodia. Presented at the 8th International Symposium on Avian Influenza, Royal Holloway, University of London. UK (2012, April 1-4).
